# Flexible Use of Spatial Frames of Reference for Object–Location Memory in Older Adults

**DOI:** 10.3390/brainsci11111542

**Published:** 2021-11-20

**Authors:** Natalia Ladyka-Wojcik, Rosanna K. Olsen, Jennifer D. Ryan, Morgan D. Barense

**Affiliations:** 1 Department of Psychology, University of Toronto, Toronto, ON M5S 3G3, Canada; rolsen@research.baycrest.org (R.K.O.); jryan@research.baycrest.org (J.D.R.); morgan.barense@utoronto.ca (M.D.B.); 2 Rotman Research Institute, Baycrest Health Sciences, Toronto, ON M6A 2E1, Canada; 3 Department of Psychiatry, University of Toronto, Toronto, ON M5T 1R8, Canada

**Keywords:** spatial memory, frames of reference, aging, MoCA, cognitive flexibility, mental representations

## Abstract

In memory, representations of spatial features are stored in different reference frames; features relative to our position are stored egocentrically and features relative to each other are stored allocentrically. Accessing these representations engages many cognitive and neural resources, and so is susceptible to age-related breakdown. Yet, recent findings on the heterogeneity of cognitive function and spatial ability in healthy older adults suggest that aging may not uniformly impact the flexible use of spatial representations. These factors have yet to be explored in a precisely controlled task that explicitly manipulates spatial frames of reference across learning and retrieval. We used a lab-based virtual reality task to investigate the relationship between object–location memory across frames of reference, cognitive status, and self-reported spatial ability. Memory error was measured using Euclidean distance from studied object locations to participants’ responses at testing. Older adults recalled object locations less accurately when they switched between frames of reference from learning to testing, compared with when they remained in the same frame of reference. They also showed an allocentric learning advantage, producing less error when switching from an allocentric to an egocentric frame of reference, compared with the reverse direction of switching. Higher MoCA scores and better self-assessed spatial ability predicted less memory error, especially when learning occurred egocentrically. We suggest that egocentric learning deficits are driven by difficulty in binding multiple viewpoints into a coherent representation. Finally, we highlight the heterogeneity of spatial memory performance in healthy older adults as a potential cognitive marker for neurodegeneration, beyond normal aging.

## 1. Introduction

As we visually experience our environment, we build mental representations of encountered spatial information, such as street signs, maps, and landmarks. The ability to process and store this spatial information is critical to the everyday functioning of humans, facilitating awareness for direction of travel, position, and orientation in the environment [1,2,3,4]. Apart from our own location in space, we rely on spatial information processing to represent object locations relative to ourselves and to other objects. In memory, these spatial representations are described through two relationships: features relative to our own position are represented in an egocentric (subject-to-object) frame of reference; and features relative to each other are represented in an allocentric (object-to-object) frame of reference. Exploratory navigation from a ground-level perspective, also called route navigation, is typically associated with an egocentric frame of reference [5,6,7]. In contrast, information in an allocentric frame of reference is categorized independently of one’s own location and echoes concepts from Tolman’s [8] cognitive map, which posits that the brain forms map-like representations of our environment. Map reading from an aerial perspective, also referred to as bird’s eye or survey view, is associated with an allocentric frame of reference [9,10].

At the behavioural level, accessing spatial representations in the mind appears effortless, but this process is a multimodal activity that engages a wide range of cognitive and neural resources [11,12,13]. Given the complex nature of these resources and their interactions, spatial representations in memory are susceptible to breakdown, including with age-related neurodegeneration [14,15,16]. In healthy older adults, structural and functional changes in brain regions implicated in the spatial domain—including the hippocampus proper [17], parahippocampus [18], and the retrosplenial cortex [19]—are associated with a decline in the ability to perceive, encode, and retrieve spatial information within frames of reference in the mind. Relative to younger adults, older adults show increased reliance on an egocentric frame of reference [20,21], even in navigational or memory contexts where this strategy may be less efficient than relying on an allocentric frame of reference [15]. Yet, age-related decline in using allocentric-based strategies may be modulated by task demands. For example, tasks that include a conceptualization of an allocentric frame of reference as a three-dimensional, landmark-to-landmark relationship, largely find pronounced deficits relative to egocentric-only navigation among healthy older adults [19,22,23], but see [24]. When tasks instead require an allocentric frame of reference, operationalized as a two-dimensional map, healthy older adults perform more comparably to younger adults [9,10]. Moreover, there is compelling evidence for interindividual variability across lifespans in the use of allocentric-based cognitive maps as a strategy for encoding spatial information [13,25].

While age-related changes in mental representations of both egocentric and allocentric frames of reference have been well-documented, there is now a growing understanding that a decline in the ability to mentally manipulate, or switch between, frames of reference may reflect a cognitive marker of neuropathology [26]. Mild cognitive impairment (MCI), commonly thought to be a preceding stage of cognitive decline towards Alzheimer’s disease, has been shown to affect the flexible use of both egocentric and allocentric frames of reference, far more extensively than healthy aging [24,27,28]. Indeed, recent approaches have sought to identify older adults who may be at risk for MCI by their ability to use mental spatial representations in service of memory and navigation [29,30,31]. Yet, the dynamic nature of spatial cues in the environment means that switching between frames of reference is often required, and this switching process among older adults, both healthy and at risk for cognitive neuropathology, complicates these approaches [27]. For example, an impairment in switching from an egocentric frame of reference to an allocentric one has been demonstrated in the context of route navigation in healthy aging [32], but it remains unclear if the same pattern of impairment would apply to spatial memory for object locations. Other studies have linked both healthy aging [33] and MCI [34] to a decline in the ability to switch from an allocentric frame of reference to an egocentric one. These studies either did not compare performance to the opposite direction of switching (i.e., egocentric-to-allocentric) [33], or they focused on a small-scale tabletop memory task, without a navigational component [34]. To our knowledge, studies to date have not compared the directionality of switching for object–location memory (i.e., allocentric-to-egocentric vs. egocentric-to-allocentric) in a large-scale environment among older adults. 

Heterogeneity among the healthy, non-clinical older adult population, both in terms of cognitive function [35,36,37] and spatial processing [38,39], suggests that aging may not uniformly impact the ability to flexibly form and access egocentric and allocentric spatial representations in the mind, though these factors have received limited exploration in a large-scale environment assessing spatial memory. The current study investigated the relationships among memory for object locations across frames of reference, cognitive status as measured by the Montreal Cognitive Assessment (MoCA) [40], and self-reported spatial ability in older adults. We used a computer-based virtual reality (VR) spatial memory task, in which older adult participants learned and recalled everyday objects within immersive environments, both from a first-person perspective (i.e., egocentric) and a map-based perspective (i.e., allocentric). We predicted that older adults would more accurately remember the locations of objects when frames of reference matched between encoding and recall, compared with when they switched from encoding to recall. While the existing literature on age-related deficits in the directionality of switching between frames of reference is mixed, by comparing egocentric to allocentric switching and allocentric to egocentric switching in a single paradigm, we can resolve inconsistencies in the spatial memory literature. Moreover, using a VR approach allowed us to measure memory for object locations to a high degree of precision, and within large-scale environments, thus enabling a closer investigation into the relationship between memory accuracy and potential modulating factors, including cognitive status and self-rated spatial ability.

## 2. Materials and Methods

### 2.1. Participants

Thirty older adult participants (*n_female_* = 22; *M_age_* = 75.82 years; *SD_age_* = 6.00; *M_education_* = 15.55 years; *SD_education_* = 2.49) were recruited as part of an ongoing study of aging at the University of Toronto and Rotman Research Institute, Baycrest Health Sciences. Inclusion criteria for all participants were as follows: fluent in English, normal or corrected-to-normal vision and hearing, and no history of neurological or psychiatric disorders. Data was collected across 2 sessions on separate days to reduce participant fatigue, with 20 participants returning for the 2nd session an average of 96 days later. Due to the ongoing COVID-19 pandemic, we were unable to reschedule the remaining 10 participants for a 2nd session, so fewer trials of the spatial memory task were included for these participants (see statistical analyses below). The spatial ability questionnaire and neuropsychological assessment (see Table 1) were administered in the first session, including the Montreal Cognitive Assessment (MoCA; *M_MoCA_* = 25.36, *SD_MoCA_* = 1.70) [40]. One participant scored below 20 on the MoCA and was therefore excluded from subsequent analyses, as per previous findings suggestions that older adults scoring ≤19 on the MoCA may already be displaying signs of dementia [41,42]. Informed consent was given by each participant before beginning the study. The study was approved by the Research Ethics Committee at both the University of Toronto and the Rotman Research Institute, respectively. 

### 2.2. Procedure and Questionnaires 

Each participant completed the MoCA, a brief demographics survey, and the neuropsychological assessment at the beginning of the first experiment session. An independent, trained experimenter administered and scored the MoCA to ensure that the experimenter conducting the virtual environment memory task was blind to the participant’s cognitive status during testing. The neuropsychological assessment included the following: Trail Making Test Trail A and Trail B [43]; Digit Symbol Substitution [44]; 1 min phonemic verbal fluency (FAS); 1 min categorical verbal fluency (Animal Naming); the Brief Visuospatial Memory Test—Revised (BVMT-R) [45]; the Rey Auditory Verbal Learning Test (RAVLT) [46] (see Table 1). Next, participants completed a spatial ability questionnaire—the Navigation Strategies Questionnaire (NSQ) [25]. The NSQ is a self-reported measure of individual preferences for strategies when navigating through space, and participants can be categorized as “scene-based” navigators (for an overall negative score) or “map-based” navigators (for an overall positive score) on the 14 items in the questionnaire. Map-based navigators are generally thought to have greater flexibility in navigation than scene-based navigators [47], with the former type of navigator demonstrating a preference for relying on an allocentric, survey-view of the environment.

### 2.3. Virtual Environment Design

The spatial memory task required participants to learn a series of environments created on the virtual reality platform OpenMaze [51], using Unity 3d (www.unity3d.com, Unity Technologies, accessed on 12 September 2018). Six environments were each designed with four distinct landmarks at each cardinal direction (i.e., North, South, etc.) to represent familiar, everyday spatial scenes (i.e., city, playground, ski resort, forest, construction site, farm) and the order of presentation of environments was randomized across participants. All environments could be seen from an allocentric 2D survey view and an egocentric 3D view, which were presented on a 15” Dell laptop screen. Each environment contained 5 unique everyday objects randomly assigned from a selected list of 30 possible objects from the Bank of Standardized Stimuli (BOSS) [52] on the basis of previously collected familiarity ratings (minimum 3.8/5) [52,53]. These objects, which were resized to have the same dimensions, were located at various coordinates within a 30 × 30 virtual square meter arena inside each environment and would rotate to face the participant’s viewing direction. The floor in each environment arena contained a grid tile pattern to aid in creating a sense of depth, but importantly, these tiles were not aligned with the positions of any object, so that counting tiles from the arena walls as a strategy was not useful. Critically, no 2 objects were located closer than 3 virtual meters from each other, and the 5 object locations were designed to be randomly dispersed across the 4 quadrants of the square arena to control for difficulty in locating each object across environments.

**Figure 1:**
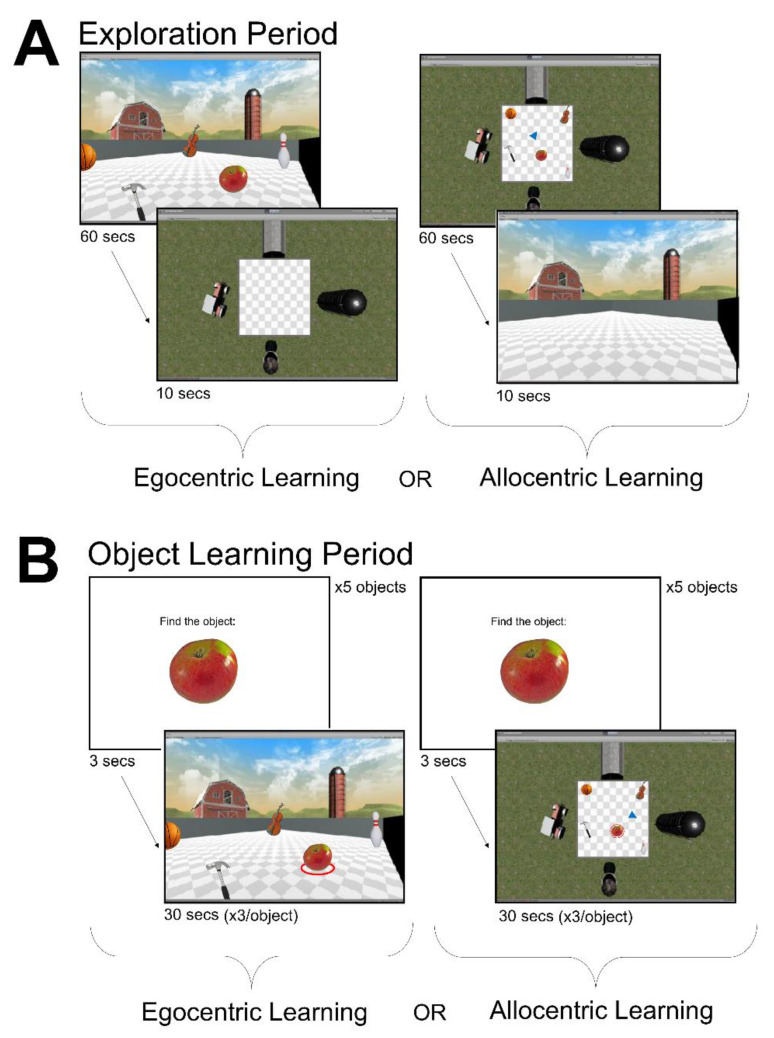
Learning phase of the spatial memory task. (**A**) In the exploration period (above) participants either saw the environment from an egocentric frame of reference or from an allocentric frame of reference for 60 s. Then, they saw the opposite frame of reference for 10 s without the objects in the arena of the environment. (**B**) In the object learning period (below) participants were shown each of the 5 objects they had to learn for that environment, and then were given 30 s to move towards the objects to find it either in the egocentric or allocentric frame of reference. The object they had to find for each trial was indicated by a red circle surrounding it (e.g., the apple in the figure above). They found each object 3 times and found a total of 5 objects per environment.

To navigate within an environment, participants used the keyboard arrow keys, with the up and down keys allowing forward and backward movements, respectively, and the right and left arrow keys allowing rotation in place. Environments presented in the egocentric frame of reference were seen by the participant from a first-person perspective, such that the laptop screen represented their field of view. Environments in the allocentric frame of reference were presented from a top–down survey perspective, with a blue arrow icon representing their position in the arena (Figure 1A). In both frames of reference, participants were limited to one direction of movement at a time (i.e., participants could not rotate and move forward simultaneously). The starting location was always in the center of the arena to minimize participants’ disorientation.

### 2.4. Spatial Memory Task

**Figure 2:**
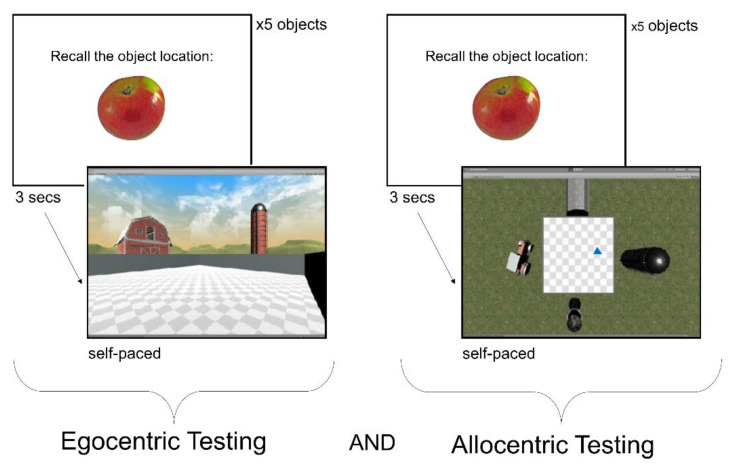
Testing phase of the spatial memory task. Participants recalled the object locations that they had previously seen in the learning phase of the spatial memory task in both the egocentric frame of reference (**left**) and the allocentric frame of reference (**right**), regardless of which frame of reference they learned the objects in. The order of frame of reference was randomized across subjects. To recall an object’s location, the participant had to move to the location where they thought they had seen the object during the learning phase in the arena of the environment.

The spatial memory task required participants to learn and recall the locations of objects within a series of virtual environments. Each learning and testing block was completed in one virtual environment (e.g., the farm environment shown in Figure 1 and Figure 2). Participants completed a minimum of 3 blocks, and up to a maximum of 6 blocks, in the 1st session, depending on their level of fatigue. A subset of participants (*n* = 20) returned for a second testing session in order to complete additional blocks. Participants completed the learning and testing phases for one environment (i.e., one block) before moving on to the next block, to minimize memory interference between environments. For each block, the environment was presented as either allocentric or egocentric during the learning phase (see Figure 1). During the testing phase, the environment was presented both as allocentric and egocentric in a randomized order within a block (see Figure 2). In other words, for each participant we were able to assess memory performance for all combinations of frames of reference in the learning and testing phases: allocentric learning–allocentric testing; allocentric learning–egocentric testing; egocentric learning–egocentric testing; and egocentric learning–allocentric testing. In this way, we were able to directly compare memory accuracy for each object location when the testing phase was in the same frame of reference as the learning phase, and in the opposite frame of reference (i.e., “switch”) from the learning phase. Participants were given a series of brief practice trials in an example environment, prior to beginning the spatial memory task.

#### 2.4.1. Learning Phase

In the learning phase of the spatial memory task, participants were first instructed to freely explore an environment for 60 s (i.e., “exploration period”) (Figure 1A). During this 60 s exploration period, they were prompted by the experimenter to move and rotate across the whole environment arena to increase familiarity with the landmarks and objects. At the end of the exploration period, participants were shown the same environment from the other frame of reference for 10 s (e.g., if the exploration period was in an egocentric frame of reference, the participant then saw the allocentric frame of reference for 10 s). This was done with the goal of improving participants’ memory accuracy from floor performance, based on initial piloting of the task. The objects within the environment arena were, however, not visible during this 10 s period. Subsequently, participants were placed back in the same frame of reference as the exploration period and given 30 s to collect each object by moving to its location in the arena (i.e., “object learning period”, see Figure 1B). For each trial of the object learning period, the object that participants were instructed to find was surrounded by a red circle. Participants repeated this step three times per object to maximize learning of their locations. During object learning period, participants were reminded to pay close attention to the object locations as they collected them.

#### 2.4.2. Testing Phase

In the testing phase of the spatial memory task, participants had to recall the locations of the objects that they had learned in each environment (Figure 2). Participants were placed in the environment arena, which no longer contained the objects they had seen during the learning phase. They would recall all five of the objects in one frame of reference and then they would recall the objects in the other frame of reference; the order presentation of the frames of reference in the testing phase was randomized for each block. Each object would be displayed for 6 s on-screen before they would see the empty environment arena. To recall an object’s location, participants were instructed to navigate to the precise location where they thought they had collected the object during the learning phase and then press the space bar to indicate their response. In the egocentric frame of reference, this required participants to move in a first-person point of view to the same location as they had been when they collected the object during the learning phase. Similarly, in the allocentric frame of reference, participants navigated a blue arrow icon in the arena to recall an object’s location and pressed the space bar to indicate their response. Participants were encouraged by the experimenter to be as precise as possible, but to take their best guess if they were unsure of their response. Note that during the testing phase, the direction that the participant (or the blue arrow icon) was facing did not matter to their response, nor did it have to match the same direction as during the learning phase.

### 2.5. Statistical Analyses

We assessed performance on the spatial memory task using a measure of Euclidean distance from each object’s presented location in the learning phase to the participant’s responses in the testing phase. This memory error measure was entered into a series of linear mixed effects models using the package *lme4* [54] in RStudio (RStudio Team, 2020; Boston, MA, USA) for R 4.0.3 (R Core Team, 2020; Vienna, Austria). Specifically, we conducted a linear mixed effects model comparing performance for trials in which the learning and testing phases were in the same frame of reference (i.e., non-switch condition) to trials in which the learning and testing phases were in a different frame of reference (i.e., switch condition). Next, we conducted two follow-up linear mixed effects models to compare the non-switching trials from learning to testing (allocentric learning to allocentric testing vs. egocentric learning to egocentric testing) and, separately, the trials that were switching from learning to testing (allocentric learning to egocentric testing vs. egocentric learning to allocentric testing), on memory error. A by-subject random intercept was included for each model to account for multiple trials in the spatial memory task per participant.

Finally, we conducted a linear mixed effects model for memory error with all four combinations of learning and testing frames of reference (i.e., allocentric learning–allocentric testing, allocentric learning–egocentric testing, egocentric learning–egocentric testing, and egocentric learning–egocentric testing) interacting with the MoCA and with the NSQ. This final model allowed us to investigate the effects of cognitive status and self-rated spatial ability on learning and testing in the frames of reference. We compared this maximal model to a depleted model (i.e., with no interaction of MoCA and NSQ with learning and testing frames of reference) using Akaike Information Criteria (AIC) as criteria for the quality of the model [55]. A significant reduction (of at least 2) in the AIC indicates that the higher model complexity (i.e., the model with added predictors) compared to the simpler model is warranted. To examine interaction effects, we used dummy coding with the allocentric learning–allocentric testing order as the fixed reference level, as this combination of learning and testing frames of reference had the lowest average memory error across participants.

## 3. Results

### 3.1. Spatial Memory Error

**Figure 3:**
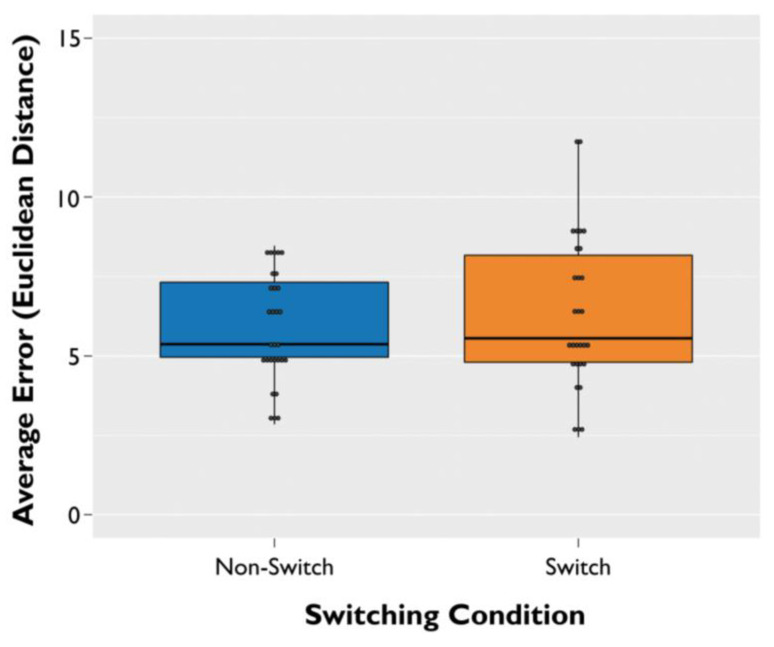
Non-switch vs. switch trial performance. Average memory error across participants for non-switch trials (i.e., same frame of reference from learning to testing phase) and switch trials (i.e., different frame of reference from the learning to testing phase). Error was calculated using Euclidean distance from the target object during the learning phase to the participant’s response during the testing phase. Error bars are ±SEM.

The linear mixed effect model for memory error showed a main effect of switching condition (β = 0.25, SE = 0.12, *t*(1002) = 2.044, *p* = 0.04), with higher memory error (i.e., worse object–location accuracy) for switch trials than non-switch trials (Figure 3).

**Figure 4:**
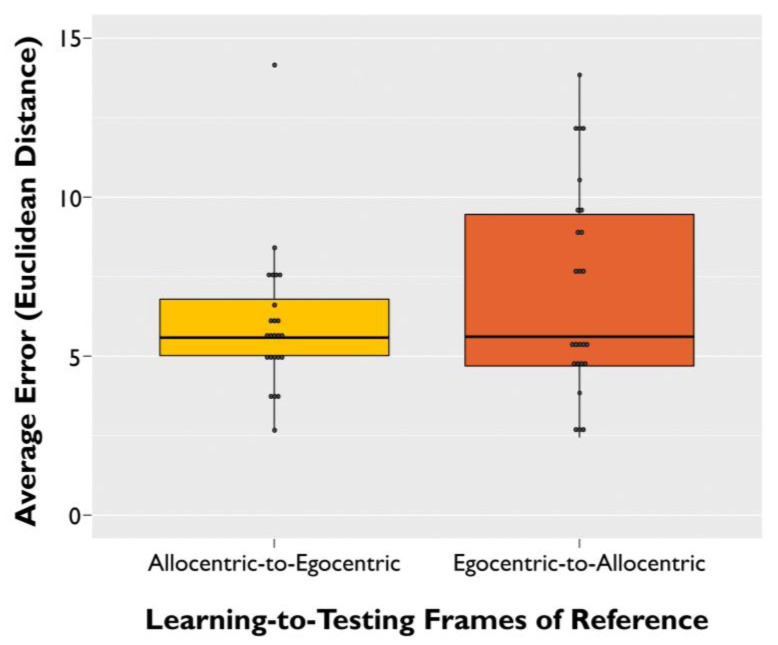
Allocentric-to-egocentric (**left**) vs. egocentric-to-allocentric (**right**) switch trial performance. Average memory error across participants for trials that required a switch between frames of reference from the learning phase to the testing phase. Average error represents Euclidean distance from the studied target object to the participant’s response during the testing phase. Error bars are +SEM.

We next examined whether the direction of switching between frames of reference from the learning phase to the testing phase (i.e., from allocentric to egocentric, or from egocentric to allocentric) differentially impacted memory error. We found a main effect of switching direction (β = 0.74, SE = 0.19, *t*(500) = 3.810, *p* < 0.001). Specifically, older adult participants had higher memory error (worse object–location accuracy) for the trials that switched from egocentric learning to allocentric testing, compared with trials that switched from allocentric learning to egocentric testing (Figure 4).

**Figure 5:**
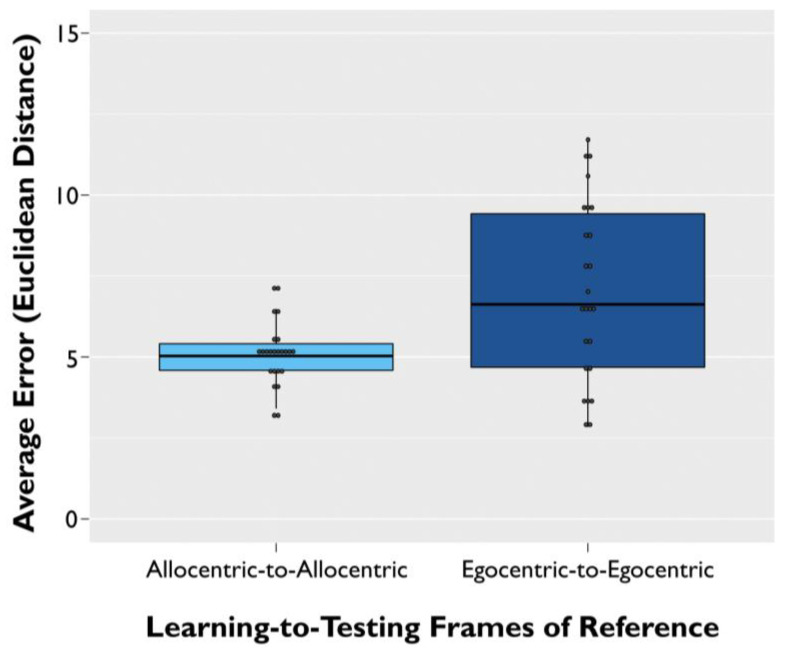
Allocentric-to-allocentric vs. egocentric-to-egocentric non-switch trial performance. Average memory error across participants for trials that did not require a switch between frames of reference from the learning phase to the testing phase. Average error represents Euclidean distance from the studied target object to the participant’s response during the testing phase. Error bars are ±SEM.

We then conducted a linear mixed effects model for memory error on trials in which the frame of reference did not switch from the learning to the testing phase. The main effect of frame of reference from learning to testing was significant (β = 1.12, SE = 0.17, *t*(502) = 6.746, *p* < 0.001). Specifically, older adult participants had higher memory error (i.e., worse object–location accuracy) for the trials learned and tested in the egocentric frame of reference over those that were learned and tested in the allocentric frame of reference (Figure 5).

### 3.2. Modulating Factors of Spatial Memory Accuracy

We investigated the relationship among the four combinations of learning-to-testing frames of reference orders (i.e., allocentric learning–allocentric testing, allocentric learning–egocentric testing, egocentric learning–egocentric testing, and egocentric learning–testing) in the task with the MoCA and NSQ, on memory error. The AIC criterion showed better model fit for the full model with interactions for the MoCA and NSQ with learning-to-testing order than the depleted null model (AIC = 5544.9, *χ*^2^(8) = 71.38, *p* < 0.001), indicating a better trade-off between goodness of fit and of model simplicity for the full model over the depleted one. We then examined interaction effects using the allocentric learning–allocentric testing order as the reference level, as this learning-to-testing order showed the lowest average memory error across participants. 

**Figure 6:**
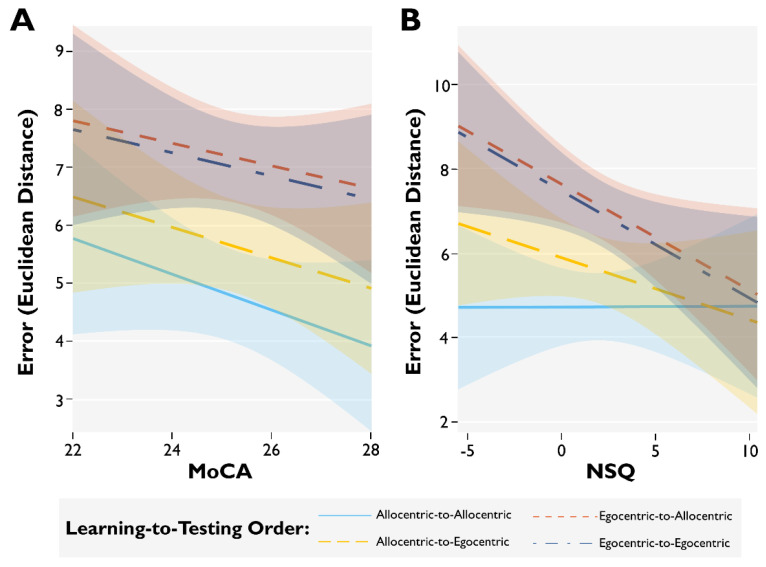
Modulatory relationships between learning-to-testing order with cognitive status and self-rated spatial ability on memory error. (**A**) The interaction effect of the MoCA with learning-to-testing order on memory error. (**B**) The interaction effect of the NSQ with learning-to-testing order on memory error. For each plot, the shaded area represents 95% CI around the fitted linear trendline and error represents the Euclidean distance from an object’s location in the learning phase to participants’ responses in the testing phase.

Compared to allocentric learning–allocentric testing, the interaction effect of the MoCA with learning-to-testing order on memory error was significant for egocentric learning–egocentric testing (β = 0.107, SE = 0.02, *t*(1002) = 6.892, *p* < 0.001), for egocentric learning–allocentric testing (β = 0.113, SE = 0.02, *t*(1002) = 7.289, *p* < 0.001), and for allocentric learning–egocentric testing (β = 0.045, SE = 0.01, *t*(1002) = 3.035, *p* < 0.01; see Figure 6A). In other words, memory error is reliably predicted by MoCA scores for all learning-to-testing orders; however, the predictive relationship between MoCA scores and egocentric learning–allocentric testing was strongest.

The interaction effect of the NSQ with learning-to-testing order on memory error was significantly greater for egocentric learning–egocentric testing (β = −0.240, SE = 0.09, *t*(1002) = −2.706, *p* < 0.01) and for egocentric learning–allocentric testing (β = −0.237, SE = 0.09, *t*(1002) = −2.664, *p* < 0.01) compared with the allocentric learning–allocentric testing reference level (see Figure 6B). The interaction of the NSQ on memory error for allocentric learning–egocentric testing did not significantly differ from the reference level of allocentric learning–allocentric testing (*p* = 0.122).

### 3.3. Exploratory Analysis of Learning Frame of Reference

We conducted two additional exploratory analyses, using linear mixed effects models, to disentangle whether the frame of reference during learning or during testing separately impacted memory error among participants, as follows: (1) a comparison of memory error during the testing phase when learning occurred in the allocentric vs. egocentric frame of reference, collapsed across frame of reference during testing; (2) a comparison of memory error during the testing phase when testing occurred in the allocentric vs. egocentric frame of reference, collapsed across frame of reference during learning. To maintain consistency with our planned models of memory error, reported above, we included a by-subject random intercept for these two exploratory models to account for multiple trials in the spatial memory task per participant.

**Figure 7:**
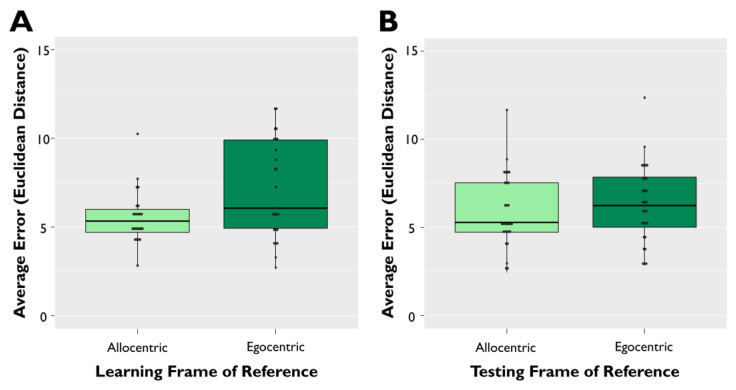
Exploratory comparison for allocentric vs. egocentric trial performance. (**A**) Average memory error during the testing phase across participants for trials learned in either the allocentric or egocentric frame of reference, regardless of testing frame of reference. (**B**) Average memory error during the testing phase across participants for trials tested in either the allocentric or egocentric frame of reference, regardless of learning frame of reference. For each plot, average error represents Euclidean distance from the studied target object to the participant’s response during the testing phase. Error bars are ±SEM.

The main effect of frame of reference during learning was significant (β = 1.93, SE = 0.26, *t*(1002) = 7.356, *p* < 0.001). Specifically, older adult participants had higher memory error during testing (i.e., worse object–location accuracy) for trials learned in the egocentric frame of reference over those that were learned in the allocentric frame of reference, regardless of the frame of reference at testing (Figure 7A). However, the main effect of frame of reference during testing was not significant (β = 0.38, SE = 0.24, *t*(1002) = 1.580, *p* = 0.114), such that memory error did not differ between trials tested in the allocentric frame of reference and trials tested in the egocentric frame of reference (Figure 7B).

## 4. Discussion

In the current study, we investigated how switching between frames of reference related to memory error for object locations among older adults in a VR spatial task. We show that older adults recalled the locations of objects less accurately (i.e., with higher memory error) when switching between frames of reference from learning to testing compared with when no switching was required from learning to testing. Our findings also provide comprehensive evidence for the directionality of this switching deficit among older adults; specifically, older adults performed worse when switching from learning in an egocentric frame of reference to testing in an allocentric one, compared with when switching from learning in an allocentric frame of reference to testing in an egocentric one. Crucially, we found that this difference in memory error for the directionality of switching between frames of reference was modulated by MoCA scores and self-rated spatial ability in older adults, such that both higher cognitive function and spatial ability resulted in better accuracy when learning occurred in an egocentric frame of reference, regardless of the frame of reference in which testing occurred. We conclude that older adults in our task benefitted from an allocentric learning advantage, but that worse performance when learning in an egocentric frame of reference was driven by individuals with lower MoCA and NSQ scores. Taken together, our results highlight that variability between older adults in cognitive status and self-rated spatial ability modulate object–location memory across frames of reference, even in the absence of clinical age-based neurodegeneration.

Consistent with our predictions, older adults performed more accurately (i.e., with lower memory error) in the spatial memory task when the frame of reference during learning matched the frame of reference during testing, compared with when the frame of reference switched between learning and testing. However, we also found that more accurate performance in both the switching and the non-switching trials was driven by lower memory error when participants learned in the allocentric frame of reference. In our task, the allocentric frame of reference could be seen from a single viewpoint, such that participants did not need to rotate their perspective to see the entire environment, its landmarks, and objects within the environment arena. In contrast, trials presented in the egocentric frame of reference required the integration of multiple viewpoints by rotating within the environment to see all its landmarks and object locations. Consistent with previous studies that conceptualized the allocentric frame of reference as a survey-view map, seen from a single viewpoint [9,10], we suggest that lower memory error for object locations in our task was due to lessened demands on binding multiple viewpoints into a coherent representation in mind [56,57]. If the allocentric frames of reference in our task were not discernable from a single viewpoint, such that seeing all the objects in the environment arena would have required a perspective shift, it is possible that memory error would have been worse [58]. Indeed, deficits in binding spatial information across multiple perspectives have been previously demonstrated in older adults [59], similar to those reported in patients with hippocampal damage [60].

This is not to say that the allocentric frame of reference in our task was globally easier or presented lower working memory demands for participants than the egocentric frame of reference; if that were the case, we would have expected intact performance on trials tested allocentrically, even when learning occurred egocentrically (i.e., egocentric learning–allocentric testing), relative to when both learning and testing occurred allocentrically (i.e., allocentric learning–allocentric testing). Instead, older adults in our task benefitted from an allocentric learning advantage—regardless of the frame of reference at testing, older adults performed better when they learned environments allocentrically than when they learned them egocentrically (Figure 7A). This advantage in the allocentric frame of reference, however, was not evidenced in all phases of the spatial task, as memory error did not differ between the allocentric and egocentric frames of reference during testing in our exploratory analysis (Figure 7B). Moreover, older adults performed with higher memory error when they were tested in an allocentric frame of reference but learned the environment in an egocentric frame of reference (Figure 4). To date, the literature on age-related deficits in the directionality of switching between frames of reference has been largely mixed [27,32,33,34], but our results are consistent with previous findings in older adults in switching from an egocentric to an allocentric strategy in the context of navigation [32,61]. We suggest that learning deficits may be reduced when older adults are first presented with an allocentric-based map of object locations during encoding, such that they do not need to perform any translational changes in the mind between frames of reference to encode object locations within a large-scale environment. Providing external allocentric-based map during encoding may be especially critical when older adults are presented with a novel environment, given that spatial disorientation in aging may be exacerbated by low familiarity with that environment [62,63]. Otherwise, we might also predict improved spatial orientation if we modelled environments in our spatial memory task on environments that are highly familiar to individual participants, thus reducing cognitive demands to form new spatial memory traces [64].

We found that higher MoCA scores, indicating relatively intact cognitive function, predicted better memory performance when participants learned in the egocentric frame of reference, even if during testing they were required to switch to an allocentric frame of reference. It is possible that older adults in our study with more intact cognitive function were better able to independently form an allocentric, map-like representation of each environment in the mind, even when presented with an egocentric frame of reference during learning [65]. In this way, older adults with higher MoCA scores, compared with older adults with lower MoCA scores, performed with similar overall accuracy in all combinations of frames of reference across learning and testing. Individuals with lower MoCA scores, on the other hand, relied on encoding object locations in the environments based on the externally available allocentric frame of reference presented during the learning phase and otherwise could not independently form a coherent map-based mental representation. Likewise, we found that higher NSQ scores (indicating greater flexibility in navigation strategy preference) also predicted better memory performance in the spatial task. This modulation was strongest when participants learned in an egocentric frame of reference and were then tested in either an egocentric or allocentric frame of reference. Previous studies among younger adults have demonstrated that relying on an allocentric “map-based” mental strategy in spatial contexts is more efficient than relying on an egocentric strategy [66], and that a preference for an allocentric strategy when navigating is associated with greater hippocampal volumes [25]. Here, our findings substantially advance this past research to include healthy older adults. Given that both MoCA scores and NSQ scores were important modulators of spatial memory performance in our study, especially when learning egocentrically and testing allocentrically, future work should seek to disentangle the relationship between cognitive function and self-rated spatial ability in older adults. Perhaps, older adults with preserved cognitive function are more likely to be individuals who are flexible in their navigational strategy, possibly due to more intact hippocampal volumes [67]. Otherwise, preserved cognitive function in aging may allow for more general cognitive flexibility, necessary for efficient spatial memory and navigation [23].

The present work provides comprehensive evidence for the directionality of age-related impairments in switching between frames of reference during an object–location memory task. Specifically, we propose that impairments in switching from an egocentric to an allocentric frame of reference are driven by difficulty in binding multiple viewpoints into a coherent spatial representation in the mind. Our findings highlight the individual variability of spatial memory performance as predicted by general cognitive function and self-rated spatial ability, in a sample of older adult participants without any clinical markers of decline beyond normal aging.

## Figures and Tables

**Table 1:** Average participant scores on the neuropsychological assessments. Average scores were compared to a normative sample percentile (%ile) of a similar age range to demonstrate broadly intact cognitive performances.

Neuropsychological Assessment	Mean Score (SD)	Compared Normative Sample
Trail Making Test		
Trails A	40.81 (8.75)	>60th %ile (75–79 years old) [48]
Trails B	100.52 (36.13)	>60th %ile (75–79 years old) [48]
Digit Symbol Substitution	44.52 (14.67)	>70th %ile (71–81 years old) [49]
Verbal Fluency		
Phonemic (FAS)	47.96 (12.30)	>80th %ile (60–79 years old) [50]
Categorical (Animals)	19.63 (5.73)	>75th %ile (60–79 years old) [50]
BVMT-R		
Learning	43.96 (12.30)	>80th %ile (70–79 years old) [45]
Delayed	8.85 (4.06)	>75th %ile (70–79 years old) [45]
RAVLT		
Total (trials I–IV) Learning	5.30 (2.09)	>75th %ile (72–79 years old) [46]
Delayed	8.00 (2.39)	>55th %ile (72–79 years old) [46]

## Data Availability

The list of object stimuli and the data set analyzed during the current study has been deposited in a public repository through the Open Science Framework, with the identifier doi:10.17605/OSF.IO/P8Q6Y.
